# Editorial and Miscellaneous

**Published:** 1862-12

**Authors:** 


### EDITORIAL AND MISCELLANEOUS.

*A First Rate Notice.*—The attention of subscribers in arrears for the JOURNAL, is directed to the *billet doux* which Dr. E. Ingals encloses to each. The large advance in prices renders the littles now necessary. "Mony a little makes a muckle" is a homely but excessively true Scoth proverb, and we trust each delinquent will bear in mind. All good bank notes received, and genuine Treasury notes not refused. Don't forget the address:

DR. E. INGALS,
Box 14, CHICAGO, ILL.

*About Fees.*—Everything else has gone up—flour and meat, groceries and dry goods. Tradesmen and journeymen, artists and day laborers all have struck for and received higher pay. That is, paper has gone down (when stamped with the representative of money impress) and gold is invisible. Things threaten to become worse before they are better—the latter pangs are yet to come—perhaps "as in the curing of a strong disease, even in the instant of repair and health the fit is strongest;" nevertheless that happy instant has not yet come, and it becomes the physician to imitate "the rest of mankind," and give another turn to the crank of prices. What, with license fees, and income tax, and tax on about every conceivable thing one uses, with extra taxes clapped on every time it is used in a little different way, we shall have to quit living unless there is union and reform in this respect. One-third at least must be added.

In this city, where prices average say forty per cent. of the Society's scale, a general co-operation might perhaps lift them within telescopic distance of the fee-bill. This city is demoralized by the unscrupulous underbidding of men whose position, at least, ought to lift them above such despicable expedients to secure and keep practice.

But in most places this evil is not present. We call, therefore, upon all respectable physicians, who rely upon their real knowledge and ability for success and not in selling themselves to the lowest bidder, to take hold of this matter in justice, not only to themselves, but to the profession at large. A little common sense, union and harmony, for the nonce at least, is all that is necessary to secure the result. No single practitioner of gentlemanly instincts wishes to victimize himself alone for the benefit of low-lived rivals.

---

*Variola—Its Nature and Treatment. A Monograph by Andrew Nebinger, M. D., Philadslphia.*—In this well written pamphlet the author urges what he denominates the "compensative nutritive" treatment of variola. It is an able plea in favor of what we have always believed the appropriate treatment of small-pox. He shows that it is not the inflammation which is to be feared, but exhaustion, to be met promptly by tonics, stimulants, and, especially, *nutritious diet.* We extract:

My practice has been in all cases of small-pox, during the initiatory or febrile and papular stages of the disease, to prescribe an antiphlogistic, medicinal, and dietetic treatment; but to abandon this form of treatment as soon as the papules begin to take upon themselves the vesicular form, and then commence a treatment which, in all its essentials shall be supporting.

In carrying out this treatment, I have not found any diet so useful, so grateful to the patient, or any which gave as little inconvenience to swallow when the mouth, fauces, and throat were studded with pustules, as a combination of eggs, milk, sugar, and ice, made in the proportion of one egg well beaten—half a pint of milk, sugar and ice in quantities suited to the taste and desire of the patient. When the necessity for stimuli has existed, as it does in all cases of the confluent variety of smallpox, I have added brandy, or what I think better, Monongahela whiskey, because for purity it can be more safely relied on, and can more readily be obtained. The quantity of this diet which I direct the patient to use in confluent smallpox is half a pint, or a tumblerful, with a large tablespoonful, or half an ounce, of Monongahela whiskey added to it, every two hours or every hour and a half *during the day and night.* I have frequently had patients to take as many as twelve eggs, three quarts of new milk, and eight ounces of whiskey daily for several consecutive days—and yet, with all this supporting and stimulating diet, this most excellent proteine or highly nitrogenized food, these poor fellows barely escaped sinking into the grave, some of them having had (notwithstanding they were so vigorously supported with food, and so actively stimulated) that peculiar feebleness and tremor which is always the unmistakable evidence of a breaking up of the vital forces—the threatenings of dissolution.

He does not, however, confine his patients to the eggs and milk, but permits use of meats and poultry; in short, any food abounding in proteine elements—urging "that in such cases to eat is to live, to abstain is to die." The blood vessels must be supplied with material to enable a vigorous and rapid maturation of the pustules and all the offices of vitality.

Although this is in entire opposition to the dicta of Wood, Watson, Gregory, West, and other standard authorities, there can at the present time, be no doubt of its physiological and practical correctness. The so-called secondary fever is not to be broken down, but sustained. It is more analogous to hectic or typhoid than to the inflammatory.

In passing, we cannot forbear to say that, precisely the same principles are applicable in the treatment of all the phlegmasiæ.

---

*Causes and Cure of Diseases of the Feet: with Practical Suggestions as to their Clothing. By C. H. Cleaveland, M. D., Cincinnati*, 1862. The author, in this pamphlet, has given a very fair *resume* of the more common affections of the feet with judicious practical directions. The closing chapter, on the proper construction of shoes, is a very practical one at the present time, and we commend it especially to those whose duty it is to provide the "grand army" with *understandings*. The book is rather for popular than professional reading, but contains much valuable information.

---

*Annual Announcement of the University of the Pacific, with the Valedictory Address of Prof. Henry Gibbons, M. D.*—We judge that Prof. G. scarcely claims to be a *progressive* in medicine, nevertheless he has afforded a very readable discourse. His doctrines find but few adherents in these degenerate times.

---

*Advance Sheets.*—We acknowledge with thanks receipt of advance sheets containing several very excellent monographs read before the N. Y. Academy of Medicine:

Isaac E. Taylor, M. D., on *Non-Shortening of the Cervix Uteri.*

J. Marion Sims, M. D., on *Vaginissums.*

A. K. Gardner, M. D., on *Amputation of the Cervix Uteri.*

Alonzo Clark, M. D., on *Albuminuria.*

B. Fordyce Barker, M. D., on *Pelvic Hæmatocele.*

M. H. Ranney, M. D., on *Epilepsy.*

E. Noeggerath, M. D., on *Inversion of the Uterus.*

Comments and extracts may be given hereafter.

---

*A Good Omen.*—It is stated that Surgeon General Hammond has cut down the regulation supply of medicines to the army *fifty per cent.* The amount thus saved is appropriated to the increase of Hospital stores, including especially more nutritious diet.

Under the energetic direction of this sagacious officer the Medical Staff of the Army is becoming wonderfully enhanced in efficiency. Abuses are rectified as soon as discovered, improvements constantly made and the range of usefulness extended even though the Gordian knot of red tape have to be severed by often repeated strokes of his trenchant official catlin.

*Physical Labor in Insanity.*—At the annual meeting of the Association of Superintendents of Insane Asylums in the U. S., it was pretty generally agreed that those patients among the males who were engaged in outdoor labor, and those among the females who employed themselves indoors were always the most likely to be cured and go home.

The pecuniary value of the labor performed does not prove very considerable, but as a remedial agent it is of very great importance, as Dr. Gray remarked, "beneficial to the patient in promoting his comfort, facilitating his recovery, and producing quiet and order throughout the house." Great care is requisite that they do not work beyond their strength or until very weary.

Practical disadvantages sometimes ensue, owing mainly to the interference of friends and others who think if the patients work they ought not to pay for their board or treatment.

The idea is coming up everywhere, that those agencies which promote digestion and the formation of *good blood* with which to bathe diseased parts *from within*, *are the best curatives in all diseases.*

---

*Cause of Parturition at a fixed Period.*—A reviewer in the Sept. No. of the *Am. Med. Monthly*, commenting at some length on Prof. Bedford's Treatise on Obstetrics, refers particularly to the Author's explanation of the reason why parturition occurs at the close of a fixed period, and endorses it by the following remark: "We, therefore, now unreservedly agree with the reported judgment of one, himself a *Magnate* in physiology," [We suppose Prof. Paine is here intended, termed a '*Magnate*' from the size of his books.—Ed.] "that the explanation is *ingenious, plausible and satisfactory;* nay, more—we believe that it will be recognized in the future as the nearest possible approximation to the truth that could have been made at this time."

Prof. Bedford's explanation is certainly an approximation to the truth, better perhaps than Avicenna's "Grace of God"

doctrine—nevertheless, it fails wholly to include cases of spontaneous, or induced, abortion or miscarriage, which should be traced to the same mechanism.

Prof. B. attributes the uterine contraction to "*a matured development of the muscular structure of the organ itself*,"—which accounts for nothing but labor at nine months.

The true doctrine as many years ago (1848) elucidated by the present writer [Allen] and which may be found as published in the *Medical Independent*, Oct., 1857, p. 449, and republished in the *Reese's Medical Gazette*, Nov. 1857, p. 685, and in very many other journals. We extract from that article:

"Why does parturition occur at a fixed period?

"Because the changes in the blood of the mother, occasion-"ed by the full development of the fœtus, produce that par-"ticular molecular change in the uterine muscle which neces-"sitates contraction. The molecular change is the cause.

"The same blood-modification may occur from accidental "causes, or through the medium of the nervous arc.'

Here it will be seen that all the phenomena are reducible to the same series. Diseases, poisons, mechanical agencies, or full development of the fœtus inducing that particular molecular change which, in all muscular structures, necessitates contraction. The mechanism is identical in all physiological, and indeed pathological, acts of extrusion.

But what puzzles us mainly is, that he of the "Institutes" should pronounce "*ingenious*, *plausible*, *satisfactory*," that which is essentially a doctrine which, in the delectable work alluded to, (§ 349 *d*) he calls upon all intellectual physicians to turn from with "LOATHING AVERSION!"

---

Surgeon J. V. Z. BLANEY, U. S. Vols., has been ordered to turn over the property in his charge (as Medical Purveyor at Alexandria, Va.) to the Medical Purveyor at Washington, and report for duty to Gen. Viele at Norfolk, Va. Surgeon I. H. RAUCH, U. S. Vols., has been directed to report to Maj. Gen. Banks for duty with General Augers' command.

Directory of Hospitals.—The attention of clergymen, editors, and others is respectfully requested to the following notice, which is of interest to all who have friends in the army, and which it is therefore desirable should be widely published:

DIRECTORY OF THE HOSPITALS.

The Sanitary Commission have established an office of information in regard to patients in the Hospitals of the District of Columbia, and of Frederick City, Maryland. By a reference to books, which are corrected daily, an answer can, under ordinary circumstances, be given by return mail to the following questions:

1st. Is —— —— [giving name and regiment] at present in the hospitals of the District or of Frederick city?

2nd. If so, what is his proper address?

3rd. What is the name of the Surgeon or Chaplain of the hospital?

4th. If not in hospital at present, has he recently been in hospital?

5th. If so, did he die in hospital, and at what date?

6th. If recently discharged from hospital, was he discharged from service?

7th. If not, what were his orders on leaving?

The Commission is prepared also to furnish more specific information as to the condition of any patient in the District hospitals, within twenty-four hours after a request to do so, from an officer of any of its corresponding societies.

The office of the Directory will be open daily from 8 o'clock a. m. to 8 o'clock p. m., and accessible in urgent cases at any hour of the night.

The number of patients in these hospitals is about 25,000. If found to be practicable, the duty here undertaken locally by the Commission will be extended to include all the general hospitals in the country.

Fred. Law Olmsted,
*General Secretary.*

Adams House, 244 F street,
*Washington, D. C., November* 19, 1862.

*The Physician's Hand-book of Practice for* 1863. *By William Elmer, M. D.*—Those who have become accustomed to the convenience of this pocket memorandum would very much miss its annual appearance. Among other things, it contains a classification of diseases, a list of remedial agents, of incompatibles, poisons and their antidotes, a diagnostic examination of the urine, a record of practice and treatment, an obstetric calendar, a general memoranda, &c. It may be obtained from our principal book-dealers, or from the Publisher, W. A. Townsend, New York.

---

*Dentition and its Derangements. A Course of Lectures by A. Jacobi, M. D., Prof. of Infantile Pathology, Therapeutics. New York : Bailliere Bros.*, 1862.—The large extracts given from this series in our pages precludes the necessity of our recommending this little work to our readers. It is a valuable contribution to the literature of the subject.

---

Overcrowding of Military Hospitals.—In the French military hospitals 1700 cubic feet of air-space are allowed by regulation to each patient. In this country the minimum is fixey at 1000 cubic feet. And yet of our 150 military hospitals, we venture the assertion that not five per cent. allow 800 cubic feet of air-space to each patient, whatever may be their system of ventilation. In the majority 700 cubic feet is the maximum of air-space to each patient, and from this point hospitals may be instanced representing various figures in the descending scale as low as 250 cubic feet. And this last amount—little better than the famous Black Hole—is the maximum of air-space allowed in hospital buildings originally constructed for barracks, and almost destitute of ventilation. In large numbers of hospitals the beds are arranged at given intervals, without the slightest regard to the cubical area of the wards. The results of overcrowding are apparent in every hospital where it is practised, in the prevalence of low forms of fever, erysipelas, &c—*American Medical Times.*

